# Improved taxonomic assignment of rumen bacterial 16S rRNA sequences using a revised SILVA taxonomic framework

**DOI:** 10.7717/peerj.6496

**Published:** 2019-03-05

**Authors:** Gemma Henderson, Pelin Yilmaz, Sandeep Kumar, Robert J. Forster, William J. Kelly, Sinead C. Leahy, Le Luo Guan, Peter H. Janssen

**Affiliations:** 1 Grasslands Research Centre, AgResearch, Palmerston North, New Zealand; 2 Microbial Genomics and Bioinformatics Research Group, Max Planck Institute for Marine Microbiology, Bremen, Germany; 3 Lethbridge Research and Development Centre, Agriculture and Agri-Food Canada, Lethbridge, AB, Canada; 4 Department of Agricultural, Food and Nutritional Science, University of Alberta, Edmonton, AB, Canada

**Keywords:** Rumen bacteria, 16S rRNA genes, Taxonomic assignment, Next generation sequencing, Working taxonomic framework, SILVA

## Abstract

The taxonomy and associated nomenclature of many taxa of rumen bacteria are poorly defined within databases of 16S rRNA genes. This lack of resolution results in inadequate definition of microbial community structures, with large parts of the community designated as incertae sedis, unclassified, or uncultured within families, orders, or even classes. We have begun resolving these poorly-defined groups of rumen bacteria, based on our desire to name these for use in microbial community profiling. We used the previously-reported global rumen census (GRC) dataset consisting of >4.5 million partial bacterial 16S rRNA gene sequences amplified from 684 rumen samples and representing a wide range of animal hosts and diets. Representative sequences from the 8,985 largest operational units (groups of sequence sharing >97% sequence similarity, and covering 97.8% of all sequences in the GRC dataset) were used to identify 241 pre-defined clusters (mainly at genus or family level) of abundant rumen bacteria in the ARB SILVA 119 framework. A total of 99 of these clusters (containing 63.8% of all GRC sequences) had no unique or had inadequate taxonomic identifiers, and each was given a unique nomenclature. We assessed this improved framework by comparing taxonomic assignments of bacterial 16S rRNA gene sequence data in the GRC dataset with those made using the original SILVA 119 framework, and three other frameworks. The two SILVA frameworks performed best at assigning sequences to genus-level taxa. The SILVA 119 framework allowed 55.4% of the sequence data to be assigned to 751 uniquely identifiable genus-level groups. The improved framework increased this to 87.1% of all sequences being assigned to one of 871 uniquely identifiable genus-level groups. The new designations were included in the SILVA 123 release (https://www.arb-silva.de/documentation/release-123/) and will be perpetuated in future releases.

## Introduction

Ruminants have a complex digestive system, and digestion of feed takes place initially in the rumen. Rumen microorganisms break down feed components such as cellulose- and hemicellulose-rich plant fiber, other carbohydrates, and proteins, producing short chain fatty acids that provide energy for the host. These microorganisms are thus essential for the host, and play a key role in the nutrition and productivity of ruminants. Understanding the function and composition of rumen microbial communities is useful for efforts that aim to understand or improve animal productivity and to reduce energy loss to methane, as well as other animal production characteristics (Weimer, 2015).

Rumen microbial communities contain >10^10^ microorganisms per gram of rumen contents (Russell & Rychlik, 2001). These belong to many different species of bacteria, archaea, ciliate protozoa, fungi, and viruses, the majority of which are yet to be cultured or characterized (Berg Miller et al., 2012; Creevey et al., 2014; Henderson et al., 2015). Analysis of taxonomically-informative marker genes allows detection and quantification of uncultivated microorganisms and is widely used to identify microorganisms associated with differences such as response to diet (Henderson et al., 2015), methane yield (Kittelmann et al., 2014), feed conversion efficiency (Carberry et al., 2012), and milk composition (Jami, White & Mizrahi, 2014). Commonly used marker genes are those for the 16S and 18S rRNAs, which may be amplified by PCR or extracted bioinformatically from metagenomic datasets (Ellison et al., 2014; Kittelmann et al., 2013; Li et al., 2016). Taxonomic identities from large sequence datasets can be efficiently inferred using streamlined analysis pipelines such as mothur (Schloss et al., 2009), QIIME (Caporaso et al., 2010), RDPipeline (Cole et al., 2014), SILVAngs (Quast et al., 2013; Klindworth et al., 2013), and STAP (Wu et al., 2008), which match new sequences generated from samples to reference sequences in taxonomic reference frameworks such as Greengenes (McDonald et al., 2012), RDP (Cole et al., 2014), and SILVA (Quast et al., 2013). Each reference sequence has a taxonomic identity associated with it, allowing the abundance of sequences affiliated with different taxa to be inferred. The criteria for inclusion and the taxonomic designations (e.g., naming conventions, taxonomic resolution) applied to reference sequences differ between taxonomic frameworks. Efforts are being made to unify naming conventions (Konstantinidis & Tiedje, 2005; Rosselló-Móra, 2012; Yilmaz et al., 2014; Yarza et al., 2014; Hinchliff et al., 2015; Parks et al., 2018), but the choice of taxonomic reference framework used to analyze a dataset has a direct impact on the results and therefore also on the ability to directly compare studies analyzed using different frameworks (Liu et al., 2008; Newton & Roeselers, 2012; Balvočiūtė & Huson, 2017; Ritari et al., 2015).

The lack of taxonomic resolution of sequence data that results from using “general purpose” frameworks has led researchers to develop “environment-specific taxonomies” to improve the taxonomic assignment at lower classification levels. This is particularly necessary for microbial systems where only a comparatively small number of microorganisms have been validly characterized and described. Examples of such custom databases include DictDb for the analysis of termite and cockroach gut microbiota (Mikaelyan et al., 2015), TaxAss for freshwater bacteria (Rohwer et al., 2018), or RIM-DB for rumen and intestinal methanogenic archaea (Seedorf et al., 2014). Overall, use of environment-specific taxonomic frameworks and reference databases significantly improves taxonomic assignment of sequence data (Ritari et al., 2015; Mikaelyan et al., 2015; Seedorf et al., 2014).

Though attempts have been made to bring more rumen bacteria into cultivation in recent years (Kenters et al., 2011; Nyonyo et al., 2013; Seshadri et al., 2018), comparatively few novel isolates have been validly described or named. This means they are unlikely to be included as named species in general purpose taxonomic frameworks, and sequences that match these organisms will be classified as “other” or “uncultured” sequences in a given phylum, class, order, or family. Furthermore, many sequences classified together at the genus level in existing frameworks display low sequence similarities, meaning their taxonomic resolution needs to be improved. Separating these out into multiple genera has been progressing since 16S rRNA gene sequence data became routinely available (e.g., Collins et al., 1994; Yutin & Galperin, 2013). Pioneering work by Kim, Morrison & Yu (2011) initiated efforts to group high-quality 16S rRNA gene sequences from rumen bacteria into genus-level clusters. However, many rumen bacterial sequences still cannot be reliably classified to the genus level, and sometimes not even at the family level, meaning there is a need to further improve the classification and resolution of rumen bacterial sequencing data. The genus level is often used for taxonomic assignment of next generation sequencing data from rumen samples, mainly because the read lengths generated are short (200 to 400 bp), making resolution to the species level unreliable depending on which region of the 16S rRNA gene is used (Kim, Morrison & Yu, 2011).

We used the bacterial 16S rRNA gene sequence dataset generated during the global rumen census (GRC) project (Henderson et al., 2015) to identify rumen bacterial groups in need of taxonomic refinement, and then refined the taxonomic designations of the most abundant taxonomic groups found in rumen samples. Here, we report our process and the incorporation of our refined nomenclature into the ARB SILVA taxonomic framework and database for further improvement in the future.

## Methods

### Rumen samples and microbial community data used in analyses

Sequencing data generated using 454 GS FLX Titanium chemistry from the 684 samples in the GRC study that generated usable sequence data for bacteria (Henderson et al., 2015), which covered a wide range of ruminant species on different diets, were used in this study. The samples were all processed for sequencing of bacterial 16S rRNA sequences as previously described (Henderson et al., 2015; Kittelmann et al., 2013). Raw sequencing reads (BioProjects PRJNA272135, PRJNA272136, and PRJNA273417 in NCBI’s Sequence Read Archive; Leinonen, Sugawara & Shumway (2011)) were processed in QIIME (Caporaso et al., 2010) using standard parameters unless indicated otherwise. The primers used generated amplicons containing the V1-V3 regions of the 16S rRNA gene (Henderson et al., 2015). During the split library process, sequences that were at least 400 bp long were retained (input arguments -l 400, -L 1000 -r–z truncate_remove), covering at least the V1 and V2 regions of the 16S rRNA gene. The resulting 4,557,252 sequencing reads were concatenated and grouped into 774,769 operational taxonomic units (OTUs) using UCLUST with a 97% similarity definition criterion. Taxonomic identities were assigned to the repset sequences in QIIME using a BLAST (Altschul et al., 1990) search against either the SILVA database version 119 (Quast et al., 2013), or version 119Rum (this study, see below), Greengenes (version 13_8; McDonald et al., 2012) or RDP training set versions 14 and 16 (obtained from http://www.mothur.org/wiki/RDP_reference_files). Data were summarized at the genus level. Additionally, taxonomic identities were assigned against RDP’s bacterial 16S rRNA gene dataset using Classifier release 11.4 (Cole et al., 2014; Wang et al., 2007) and a confidence cut-off of 80% to summarize data. The number of sequence reads assigned to each OTU was used to assign abundance to each repset sequence.

### Improving the resolution of the SILVA taxonomic framework

Operational taxonomic units that contained at least 50 sequences at the 97% similarity cut-off were selected for further analysis (i.e., 8,985 or 1.16% of all OTUs, representing 2,833,335 or 62.2% of all GRC sequences). These representative OTU sequences were aligned with SINA version 1.2.11 (Pruesse, Peplies & Glöckner, 2012) using the SILVA SSURef database version 119 as a reference alignment. This contained 16S rRNA (gene) sequences ≥1,200 nt. The aligned representative OTU sequences were imported into ARB version 6.0.2 (Ludwig et al., 2004), together with information on their abundance and prevalence in rumen samples, and they were added to the guide tree using the inbuilt *Escherichia coli* filter for positions 1,044–11,892 (corresponding to *E. coli* 16S rRNA gene positions 28–514). The SILVA databases and associated guide trees are rigorously curated, and sequence quality inclusion criteria, guide tree construction, and maintenance are described in detail elsewhere (Pruesse et al., 2007; Glöckner et al., 2017). The 16S rRNA gene sequences of isolates included for genome sequencing in the Hungate1000 project (Seshadri et al., 2018) were also aligned and added to the guide tree as described above. We assigned taxonomic designations down to genus-equivalent level, where possible, to all predefined monophyletic bacterial groups in the guide tree that were found to contain rumen bacterial sequences. We used the following similarities between sequences in a monophyletic radiation to define a genus-level (>94.5%) or family-level (>86.5%) group (Yarza et al., 2014). Clusters that did not also contain sequences of described species were named to the lowest taxonomic level that could be assigned and a strain, clone, or uncultured genus-level group (UCG) identifier included in the name as appropriate (e.g., *Bacteria*; *Bacteroidetes*; *Bacteroidia*; *Bacteroidales*; *Prevotellaceae*; *Prevotellaceae* NK3B31 group or *Bacteria*; *Firmicutes*; *Clostridia*; *Clostridiales*; *Ruminococcaceae*; *Ruminococcaceae* UCG_001). Taxonomic designations were independently reviewed by three of the authors, and once agreed upon by all were incorporated into the SILVA 119Rum database used for testing (see below), and then incorporated into SILVA SSURef database release 123 (http://www.arb-silva.de/documentation/release-123/).

### Assessing the taxonomic assignments generated by different taxonomic frameworks

The 50 most abundant and 50 most prevalent bacterial OTUs were used as a test subset to compare the differences in taxonomic assignments made using different frameworks. Abundance was defined as the average proportion of an OTU in all 684 samples. Prevalence was defined as the proportion of the 684 samples that the OTU occurred in. This subset contained 77 unique OTUs because some were both abundant and prevalent. Sequence alignments of these OTUs and the closest type strain and other cultured relatives based on sequence similarity were manually curated and used to generate similarity matrices. Sequence similarity cut-offs recommended by Yarza et al. (2014) were used to identify likely identities of OTU sequences at genus (94.5%), family (86.5%), order (82.0%), class (78.5%), and phylum (75.0%) levels, as these provide a unified basis for the classification and nomenclature of uncultured bacteria that is compatible with the taxonomy of cultured bacteria (Yarza et al., 2014).

## Results

### Revising the nomenclature of genus-level clusters for rumen bacteria

We first identified the radiations of abundant rumen bacteria using the SILVA 119 framework. To do this, we placed the repset sequences from the 8,985 largest OTUs, that is, those with the greatest number of sequence reads assigned to them, from the GRC dataset into the SILVA 119 tree. These fell into 241 pre-defined clusters in the guide tree provided with the SILVA 119 database. Later (see below), when the entire GRC dataset was reanalyzed after these clusters had been given unique taxonomic identifiers (again, see below), 97.8% of all sequences in the GRC dataset were assigned to these 241 clusters, showing that these potentially covered a large part of rumen bacterial diversity (Table S1).

Next, we examined these clusters based on only the sequences in the SILVA 119 database, that is, excluding the short reads from the GRC dataset. These clusters therefore contained 16S rRNA (gene) sequences ≥1,200 nt. Of these 241 clusters, 99 had no unique or had inadequate taxonomic identifiers in the SILVA 119 framework. For example, there were 12 distinct radiations named “*Lachnospiraceae* incertae sedis,” 16 named “*Ruminococcaceae* uncultured,” and three named “*Coprococcus*” (Table S1). This means that sequences matched to them would be assigned to an undefined or unclassified group at higher than the genus level, or they would be given a genus assignment that would be incorrect because the named reference sequence was >5.5% different to that of the type species of the genus. Here, we applied the genus-level sequence to sequence similarity cut-off of 94.5% identity proposed by Yarza et al. (2014). Each of these 99 clusters was given a unique nomenclature based on named species in the cluster, current trivial names used in the literature, or the names of isolates or other sequences in the cluster. Based on the level of sequence similarity and the level of separation from other named groups, these were provisionally given genus- or family-level status. Examples include the definition of one of many radiations designated as “*Lachnospiraceae* uncultured” as *Lachnospiraceae* group NK4B4, named after an isolate that falls into this genus-level group, and the distinction of multiple radiations previously all designated as “*Erysipelotrichaceae* uncultured” as *Erysipelotrichaceae* UCG001 to *Erysipelotrichaceae* UCG010, where UCG indicates a genus-level cluster without recognized cultured isolates (Table S1).

The names of an additional 11 clusters (containing 13.9% of GRC sequences) were modified to clarify their taxonomic positions, but the original names were unique and so these changes had no material impacts on the classification of sequences assigned to them. No changes were made to the nomenclature of the remaining 131 clusters, containing 20.1% of GRC sequences. Where a group contained named isolates that were clearly not in the same genus-level group as the type species of the genus, the genus name was retained but written in square brackets to indicate this. An example is the [*Eubacterium*] *ruminantium* group (Table S1), which is in the family *Lachnospiraceae*, and not close to the type species of the genus *Eubacterium*, *E. limosum*, which is in the family *Eubacteriaceae* (Ludwig, Schleifer & Whitman, 2009).

All of these new taxon designations were linked to sequences available in the SILVA SSURef database release 123 (SILVA 123 framework, http://www.arb-silva.de/documentation/release-123/). A summary of these designations is given in Table S1.

### Comparison of taxonomic assignments generated using different taxonomic frameworks

**Figure 1:**
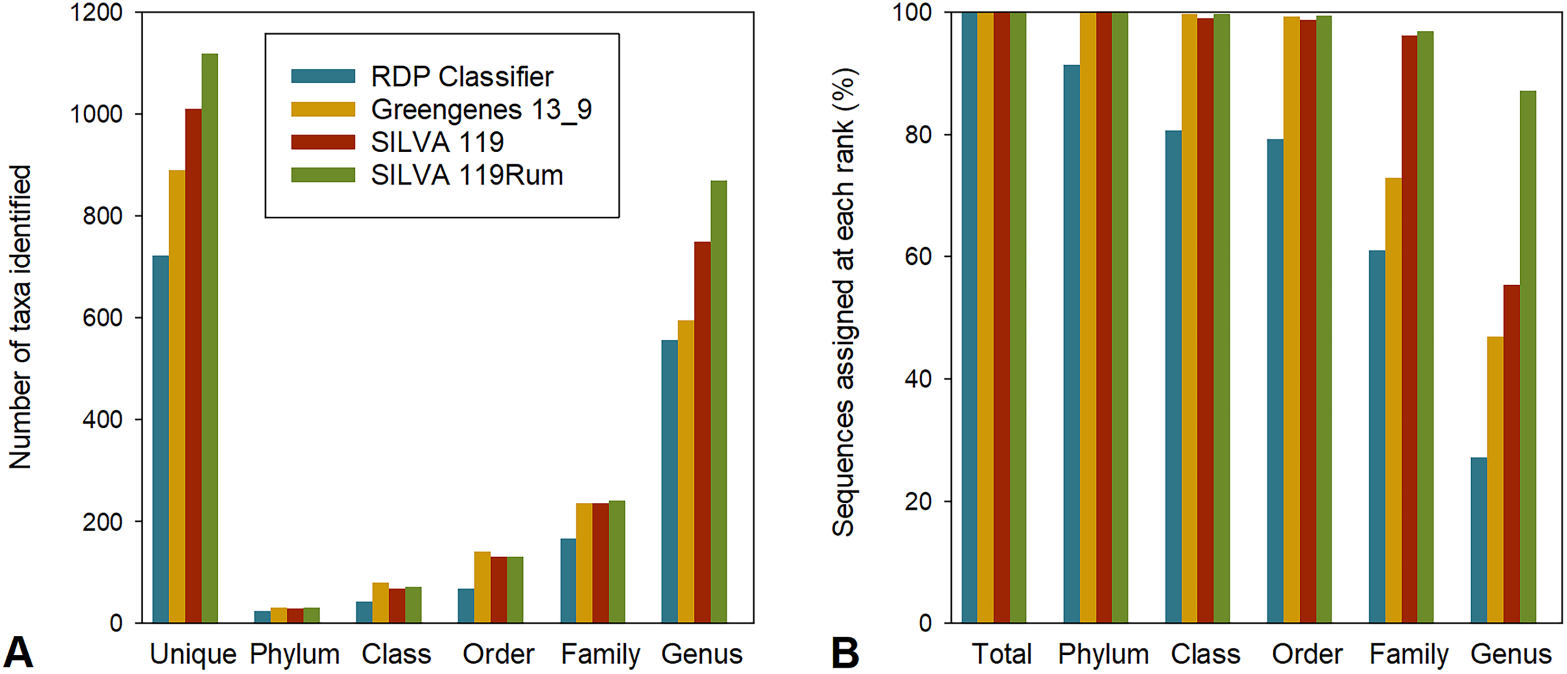
Assignment to different taxonomic ranks with different frameworks. (A) Number of taxa identified at different taxonomic ranks in the GRC dataset using different taxonomic frameworks. Also shown are the numbers of unique taxonomic strings returned. (B) Assignment (%) of GRC sequences to defined taxa at different taxonomic ranks using different frameworks.

To allow us to assess the impact of the improved taxonomic resolution, we produced a temporary version of SILVA119 that included these new designations, which we called SILVA 119Rum. We compared the taxonomic assignments made using this new framework with those made using four other frameworks, namely Greengenes 13_8, RDP release 11.4 (using RDP Classifier), RDP training sets 14 and 16, and the parent, SILVA 119. For our comparisons, we used all 774,769 OTUs generated from the 4,557,252 partial bacterial 16S rRNA gene sequences that had been generated from 684 rumen samples in the GRC dataset, rather than just the 8,985 largest OTUs that we used to identify the bacterial groups that needed refinement. The repset sequences from this larger set of OTUs were assigned to between 723 and 1,441 uniquely taxonomic strings using the different frameworks (Fig. 1A), although not all were resolved to the genus level (Fig. 1B). The proportion of sequences in the GRC dataset that were assigned to a taxon at any rank that was uniquely labelled within the preceding rank was also calculated (Fig. 1B). For example, genus-level groups labelled uncultured or incertae sedis without further qualifiers were not considered unique, since it is not possible to tell how many genus-level groups with the same identifier there might be in a family. There were between 557 and 1,355 identifiable genus-level taxa (Fig. 1A) using the different frameworks.

Overall, assignments made using RDP release 11.4 were the most conservative, returning the smallest number of taxa (Fig. 1A) and assigning the lowest proportion of sequences to a taxon at any taxonomic rank (Fig. 1B). The Greengenes taxonomic framework resulted in the second most conservative assignment of taxonomic identities to sequences, followed by the SILVA 119 and SILVA 119Rum taxonomic frameworks. Greengenes and RDP release 11.4 resulted in fewer <50% and <30% of sequences being assigned to a named genus, respectively. The SILVA 119 taxonomic frameworks allowed 55.4% of sequence data to be assigned to 751 uniquely-named genera. The improved SILVA 119Rum framework resulted in 87.1% of all sequences in the GRC dataset being assigned to one of 871 uniquely identifiable genus-level groups.

The RDP training sets (RDP versions 14 and 16) resulted in >95% of sequences being assigned at the genus level when they were used as BLAST databases (Table S2). The RDP training sets predominantly contain sequences of characterized organisms whose taxonomic strings are nearly all resolved to the genus level. It contains few reference sequences from unnamed or taxonomically poorly-resolved groups. For this reason, these frameworks are not suitable for use with the QIIME “parallel_assign_taxonomy_blast.py” script. This script always assigns a match to a sequence, and by default that match will almost always return a genus name because the RDP training sets contain few sequences without a valid genus name. Sparse databases like the RDP training sets will result in an assignment to a match that may not be a close one. This results in an over-assignment of sequences to named genera to which the sequences do not belong; however, if these databases were used with Classifier as intended, it is likely these assignments would have been given low confidence scores (Wang et al., 2007) that would not result in a genus level assignment, as we observed (Fig. 1A).

### Comparison of apparent microbial community composition using different frameworks

**Figure 2:**
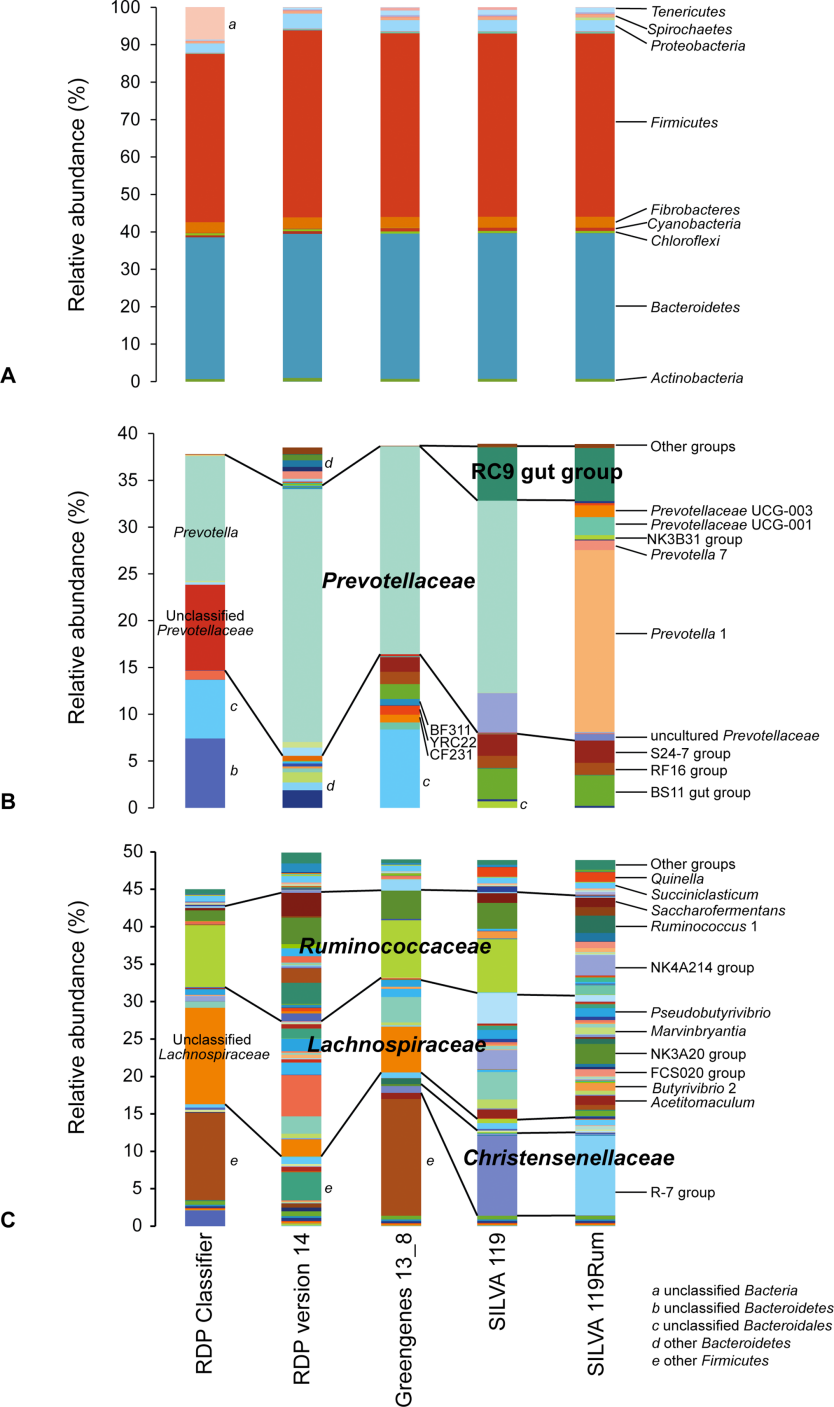
Schematic showing relative abundances of taxa after assignment of sequences using different taxonomic frameworks. (A) shows assignments at the phylum level. (B) and (C) show taxa affiliated with the phyla (B) *Bacteroidetes* and (C) *Firmicutes* that occur at an abundance of at least 0.05% in any one sample. Any genus level taxa with a relative abundance below 0.05% are grouped together as “Other groups.” Blocks with the same colors represent the same taxonomic designations in the different frameworks. More detailed microbial community compositions are provided in Table S3.

Apparent rumen microbial community compositions were broadly comparable at phylum level, regardless of the taxonomic framework used (Fig. 2A). However, at the genus level the apparent make-up within the dominant phyla *Bacteroidetes* (Fig. 2B) and *Firmicutes* (Fig. 2C) differed considerably. This was due to the different naming conventions, different nomenclature, and finer taxonomic resolution of groups in some of the databases. This means that data analyzed using different frameworks cannot be directly compared, as can be seen in Figs. 2B and 2C, where the same dataset gave very different apparent community structures using the different taxonomic frameworks. The impact of these differences is illustrated by the taxonomic identities assigned to individual OTUs using the different taxonomic frameworks. As examples, we show the taxonomic assignments of the 77 most abundant and prevalent OTUs in the GRC dataset made using the different frameworks (selected data are shown in Table 1; full data in Table S2). In some cases, the differences were in nomenclature, but in others it was due to the lack of resolution near the genus-level. Other examples of the taxonomic equivalence of groups with different names in the different frameworks are shown in Fig. 2.

**Table 1:** Comparison of the nomenclature of taxonomic assignments made using different databases.

OTU ID	Average abundance (%)	RDP release 11.4	Greengenes 13_8	SILVA 119	SILVA 119Rum
365725	0.240	g_*Succinivibrio*	f_*Succinivibrionaceae*	g_*Succinivibrio*	g_*Succinivibrio*
722152	0.558	c_*Gammaproteobacteria*	f_*Succinivibrionaceae*	f_*Succinivibrionaceae*	g_*Succinivibrionaceae* UCG_001
15480	0.265	c_*Gammaproteobacteria*	f_*Succinivibrionaceae*	f_*Succinivibrionaceae*	g_*Succinivibrionaceae* UCG_002
9138	0.231	g_*Ruminobacter*	g_*Ruminobacter*	g_*Ruminobacter*	g_*Ruminobacter*
142948	0.556	f_*Prevotellaceae*	g_*Prevotella*	g_*Prevotella*	g_*Prevotella* 1
664059	0.243	g_*Prevotella*	g_*Prevotella*	g_*Prevotella*	g_*Prevotella* 7
480108	0.475	g_*Fibrobacter*	g_*Fibrobacter*	g_*Fibrobacter*	g_*Fibrobacter*
90393	0.504	o_*Clostridiales*	o_*Clostridiales*	f_*Christensenellaceae*	g_*Christensenellaceae* R-7 group
284365	0.269	g_*Succiniclasticum*	g_*Succiniclasticum*	g_*Succiniclasticum*	g_*Succiniclasticum*
237285	0.407	g_*Pseudobutyrivibrio*	g_*Pseudobutyrivibrio*	g_*Pseudobutyrivibrio*	g_*Pseudobutyrivibrio*
301314	0.051	g_*Butyrivibrio*	g_*Butyrivibrio*	g_*Butyrivibrio*	g_*Butyrivibrio* 2
493059	0.348	f_*Lachnospiraceae*	g_*Butyrivibrio*	g_*Butyrivibrio*	g_*Lachnospiraceae* NK3A20 group
698124	0.265	f_*Lachnospiraceae*	f_*Lachnospiraceae*	g_*Acetitomaculum*	g_*Acetitomaculum*
732718	0.145	f_*Lachnospiraceae*	g_*Blautia*	g_*Blautia*	g_*Blautia*
109054	0.049	f_*Lachnospiraceae*	g_*Moryella*	g_*Moryella*	g_*Moryella*
234051	0.067	f_*Lachnospiraceae*	g_*Coprococcus*	f_*Lachnospiraceae*	g_[*Eubacterium*] *ruminantium* group
311462	0.060	f_*Lachnospiraceae*	o_*Clostridiales*	f_*Lachnospiraceae*	g_[*Ruminococcus*] *gauvreauii* group
205298	0.077	f_*Lachnospiraceae*	o_*Clostridiales*	f_*Lachnospiraceae*	g_*Lachnospiraceae* FCS020 group
605934	0.068	f_*Lachnospiraceae*	f_*Lachnospiraceae*	f_*Lachnospiraceae*	g_*Lachnospiraceae* AC2044 group
295461	0.148	f_*Ruminococcaceae*	f_*Ruminococcaceae*	f_*Ruminococcaceae*	g_[*Eubacterium*] *coprostanoligenes* group
401207	0.124	f_*Ruminococcaceae*	f_*Ruminococcaceae*	f_*Ruminococcaceae*	g_*Ruminococcaceae* NK4A214 group
237588	0.070	o_*Clostridiales*	f_*Ruminococcaceae*	g_*Ruminococcus*	g_*Ruminococcus* 1
580981	0.140	f_*Ruminococcaceae*	g_*Ruminococcus*	g_*Ruminococcus*	g_*Ruminococcus* 2
139212	0.180	f_*Ruminococcaceae*	f_*Ruminococcaceae*	g_*Saccharofermentans*	g_*Saccharofermentans*

**Note:**

Shown are the 24 unique examples among the 25 most abundant OTUs and 10 most abundant *Lachnospiraceae* and *Ruminococcaceae*, listing the lowest defined rank with a unique identifiable name. Each name is preceded by a letter giving the rank: c = class, o = order, f = family, g = genus. The average abundance in the GRC dataset is also given. The full dataset of 77 OTUs is given in Table S2.

### Increased taxonomic resolution with SILVA 119Rum

More OTUs, and hence more sequences otherwise assigned only to the family level (such as *Lachnospiraceae*, *Ruminococcaceae,* and *Succinivibrionaceae)*, were assigned to multiple new genus-level groups within these families using the SILVA 119Rum framework (Table 2). Other frameworks only assigned these OTUs to order or family levels, or to genera that are not monophyletic or contain sequences with sequences that have <5.5% identity. These finer scale subdivisions of families and genera are reflected in the greater number of genus-level taxa found in the GRC dataset classified using SILVA 119Rum (Figs. 1A, 2B and 2C).

**Table 2:** Assignment of sequences in the GRC dataset to bacterial families and to named genera within those families.

Family	Assigned	RDP release 11.4	Greengenes 13_8	SILVA 119	SILVA 119Rum
*Prevotellaceae*	To genera in family	13.76 (5)	22.20 (1)	20.61 (3)	24.80 (13)
	To family	22.92 (6)	22.42 (2)	24.95 (5)	24.96 (15)
*Christensenellaceae*	To genera in family	0 (0)	0 (0)	0.05 (1)	10.64 (1)
	To family	0 (0)	0.93 (1)	10.70 (2)	10.69 (3)
*Lachnospiraceae*	To genera in family	2.01 (25)	6.49 (20)	10.24 (27)	15.95 (69)
	To family	15.56 (27)	12.59 (21)	16.90 (30)	16.98 (72)
*Ruminococcaceae*	To genera in family	2.31 (21)	3.97 (7)	5.22 (18)	13.11 (43)
	To family	10.59 (22)	11.64 (8)	13.25 (21)	13.17 (45)
*Succinivibrionaceae*	To genera in family	0.76 (4)	0.63 (5)	0.75 (5)	1.78 (7)
	To family	0.83 (5)	1.78 (6)	1.80 (6)	1.80 (8)

**Note:**

The numbers are the average percentage that those sequences make up in samples in the GRC dataset. The numbers in parentheses are the number of genera to which the sequences are assigned or the number of groups within the family (these include subgroups designated as “unclassified” and “uncultured” that have no unique genus-level identifier).

The great diversity of rumen *Lachnospiraceae* and *Ruminococcaceae* that is not yet formally described and named has been reported previously (Creevey et al., 2014). Sequences from members of *Lachnospiraceae* made up >17% of the GRC dataset. Refinement of the taxonomy increased the genus level separation, with three quarters of those sequences assigned to more exact genus-level taxa (Table 3; Table S1). This included separation of undefined taxa named *Lachnospiraceae* incertae sedis and uncultured *Lachnospiraceae*. Named genera like *Butyrivibrio*, *Blautia*, and *Coprococcus* also appeared to contain sequences originating from multiple genus-level groups. Similarly, *Ruminococcaceae* contained >13% of all GRC sequences, and over half of these were classified in SILVA 119 as *Ruminococcaceae* incertae sedis or as uncultured *Ruminococcaceae*. These were separated into 20 new genus-level groups, one of which, *Ruminococcaceae* NK4A214 group, contained nearly 2.8% of all GRC sequences (Table S1). This group is named after isolate NK4A214, the first recognized isolate that belongs in this apparently new genus (Kenters et al., 2011).

**Table 3:** Genus-level taxa in the family *Lachnospiraceae*.

SILVA 119		SILVA 119Rum	
Taxon	Abundance (%)	Abundance (%)	Taxon
*Acetitomaculum*	1.194	1.194	*Acetitomaculum*
*Blautia*	1.574	0.558	*Blautia*
		1.016	*Lachnospiraceae* NK4A136 group
*Butyrivibrio*	4.197	1.479	*Butyrivibrio* 2
		2.718	*Lachnospiraceae* NK3A20 group
*Coprococcus*	0.331	0.161	*Coprococcus* 1
		0.139	*Coprococcus* 2
		0.030	*Coprococcus* 3
*Dorea*	0.098	0.098	*Dorea*
*Lachnospiraceae* incertae sedis	2.552	0.031	(*Clostridium*) *aminophilum* group
		0.017	(*Clostridium*) *herbivorans* group
		0.260	(*Clostridium*) *phytofermentans* group
		0.013	(*Clostridium*) *saccharolyticum* group
		0.065	(*Eubacterium*) *cellulosolvens* group
		0.313	(*Eubacterium*) *hallii* group
		0.267	(*Eubacterium*) *oxidoreducens* group
		0.701	(*Eubacterium*) *ruminantium* group
		0.173	(*Eubacterium*) *ventriosum* group
		0.651	(*Ruminococcus*) *gauvreauii* group
		0.015	*Lachnospiraceae* UCG 004
		0.046	*Lachnospiraceae* UCG 005
*Lachnospira*	0.099	0.099	*Lachnospira*
*Marvinbryantia*	0.503	0.503	*Marvinbryantia*
*Moryella*	0.163	0.163	*Moryella*
*Oribacterium*	0.344	0.344	*Oribacterium*
*Pseudobutyrivibrio*	1.355	1.355	*Pseudobutyrivibrio*
*Roseburia*	0.610	0.610	*Roseburia*
*Shuttleworthia*	0.125	0.125	*Shuttleworthia*
*Syntrophococcus*	0.198	0.198	*Syntrophococcus*
*Lachnospiraceae* uncultured	3.692	0.965	*Lachnospiraceae* AC2044 group
		0.281	*Lachnospiraceae* FCS020 group
		0.016	*Lachnospiraceae* FE2018 group
		0.049	*Lachnospiracea*e NC2004 group
		0.389	*Lachnospiraceae* ND3007 group
		0.291	*Lachnospiraceae* NK4B4 group
		0.052	*Lachnospiraceae* UCG 001
		0.102	*Lachnospiraceae* UCG 002
		0.012	*Lachnospiraceae* UCG 003
		0.138	*Lachnospiraceae* UCG 006
		0.020	*Lachnospiraceae* UCG 007
		0.311	*Lachnospiraceae* UCG 008
		0.087	*Lachnospiraceae* UCG 009
		0.979	*Lachnospiraceae* XPB1014 group

**Note:**

The taxa are grouped so that the finer divisions using SILVA 119Rum are lined up alongside the original divisions made using SILVA 119. The abundances are the averages in the GRC dataset.

We divided some genera, such as *Prevotella* and *Ruminococcus*, into multiple genus-level groups based on their degree of sequence divergence. Sequences assigned to *Ruminococcus* were split between two different genus level groups, *Ruminococcus* 1, characterized by *R. flavefaciens* and *R. albus* and containing 67% of all *Ruminococcus* sequences in the GRC dataset, and *Ruminococcus* 2, which contains *R. bromii* and 33% of all *Ruminococcus* sequences in the GRC dataset (Fig. 2C; Table S1). The potential separation of *R. bromii* from the species that fall into *Ruminococcus* 1 was suggested by an early 16S rRNA gene sequence analysis of this genus (Rainey & Janssen, 1995).

The genus *Prevotella* contained 22.0% of all sequences in the GRC dataset when originally analyzed (Henderson et al., 2015). Finer scale subdivision of this taxon into genus-level groups still resulted in 18.3% of all sequences falling into a genus we designated *Prevotella* 1, which contained the type species of the genus, *P. melaninogenica*, and also *P. ruminicola*. Four additional genus-level groups of sequences that previously fell into *Prevotella* were identified and given unique designations: *Prevotella* 2, *Prevotella* 6, *Prevotella* 7, and *Prevotella* 9. Six further genus-level clusters, all previously named “uncultured,” were given unique designations, and so increased the resolution within the family *Prevotellaceae* (Fig. 2B; Table S1).

Other groupings defined in the SILVA 119Rum framework allowed assignment of sequences to taxa that were not included in the other databases. The RC9 gut group was absent from the Greengenes and RDP release 11.4 frameworks, meaning this group, making up on average 5.7% of sequences, was only identified when the SILVA 119 or SILVA 119Rum databases were used. Otherwise they were assigned to the order *Bacteroidales*. Members of the family *Christensenellaceae* were prominent in the GRC dataset, making up 10.7% of all sequences when reanalyzed using the SILVA 119Rum framework. This group was poorly resolved until a cluster containing isolate R-7 was defined and given a unique identifier (*Christensenellaceae* R-7 group) in SILVA 119Rum (Table 2). This genus-level group contained 10.6% of all GRC sequences and 99.5% of all of the sequences that were assigned to the family *Christensenellaceae*. In some cases, sequences that were previously assigned to poorly-defined groups were added to taxa that had names, because the reference sequences they matched to were given the same family-level designation. Examples include the family-level *Bacteroidales* BS11 gut group and S24-7 group.

## Discussion

In the initial study of the GRC, nearly half (45.8%) of all sequences in the dataset could not be assigned to a named genus (Henderson et al., 2015). The four most abundant of these groups in the GRC dataset were unclassified *Clostridiales* (15.3% of all sequences in the GRC dataset), unclassified *Bacteroidales* (8.4%), unclassified *Ruminococcaceae* (7.9%), and unclassified *Lachnospiraceae* (6.3%), which together accounted for 37.9% of the GRC dataset. The single largest named genus in the GRC dataset was the genus *Prevotella*, containing 22.0% of all sequences. The monophyletic radiation of *Prevotella* spp. that contains the type species *P. melaninogenica* has 16S rRNA sequences with average similarities of 90.6% (with similarities as low as 72.4%). This indicates that it may contain multiple genera, if a similarity threshold of 93% (Kenters et al., 2011) or 94.5% (Yarza et al., 2014) is applied. These similarity thresholds may not be applicable to this genus, and 16S rRNA gene sequence differences may not reflect phenotypic diversity (Achenbach & Coates, 2000). However, until the genus is thoroughly revised, some definition within it might uncover differences between samples that are masked when 22% of all sequences fall into one genus-level group. Similarly, the use of a 93% or 94.5% similarity threshold for other poorly-defined genera, or genus-level clusters without cultured representatives must be regarded as a temporary criterion until more is known about these radiations of bacteria. Overall, there is a need to further describe and classify novel and poorly characterized bacteria into appropriate taxa with validly published names to obtain a better understanding of the true diversity and nature of rumen microorganisms. Ideally, this should include isolation of representative strains in pure culture and their physiological characterization in combination with genome sequencing.

To improve the taxonomic assignment of sequences to identifiable groups at the genus level, we refined the nomenclature associated with bacterial 16S rRNA gene sequences from the rumen or from ruminal isolates in the SILVA 119 framework (Quast et al., 2013). We did this because the genus-level is a taxonomic rank that may be more likely to include organisms that share similar functional or structural features (Philippot et al., 2010) compared to higher taxonomic ranks, and so is a useful level to compare differences in community structure. The refinements that we developed were included in the release of SILVA that followed 119, namely SILVA 123, and are currently in the latest version, SILVA 132.

The parent SILVA 119 framework allowed 55.4% of the sequence data to be assigned to 751 uniquely identifiable genus-level groups, which was greater than when using RDP Classifier and Greengenes. The improved resolution of the taxonomy of rumen bacteria in the SILVA 119Rum framework increased this to 87.1% of all sequences being assigned to one of 871 uniquely identifiable genus-level groups. The new designations must be considered to be pragmatic decisions based on a desire to name these clusters for use in microbial community profiling. These designations have no formal standing in the taxonomic literature. Accurate taxonomic and nomenclatural decisions will rely on far more detailed polyphasic study of the organisms in these clusters. They have been defined purely to allow a better naming resolution of rumen bacterial community sequence data when they are grouped phylogenetically. Further refinements may also allow better taxonomic definition of genomes assembled from metagenomic data (if these have 16S rRNA genes associated with them), which in turn will allow better assessment of likely metabolic functions of uncultured taxa of rumen bacteria.

## Conclusions

The refined framework of nomenclature for 16S rRNA gene sequences from rumen bacteria developed here should be useful for investigating rumen microbial community structure. It provides a better separation of some of the large undefined catch-all groups above genus level by applying unique names to radiations that were previously not individually identifiable. We expected that the interim designations developed here will gradually be replaced by valid Linnaean nomenclature as these bacteria are systematically described.

## Supplemental Information

10.7717/peerj.6496/supp-1 Supplemental Information 1 Taxonomic strings of rumen bacterial groups as they were in SILVA 119 (“tax_slv” string in SSURef_NR99_119), and what they were after refinement in SILVA 123 (“tax_slv” string in SSURef_NR99_123). Also given are the average abundance, prevalence (presence in samples), and maximum abundance (in any single sample) of sequences in 684 samples in the GRC dataset that were assigned to each taxon using the SILVA 123 framework. Prevalence of an OTU is how many rumen samples it appears in (*n* = 684).

10.7717/peerj.6496/supp-2 Supplemental Information 2 Extended data for Table 1. Prevalence of an OTU is in how many rumen samples it appears (*n* = 684).

10.7717/peerj.6496/supp-3 Supplemental Information 3 Taxonomic assignment of sequences in the GRC dataset to taxa using five different taxonomic frameworks.

## Additional Information and Declarations

### Data Availability

The following information was supplied regarding data availability: The revised taxonomic affiliations are included in SILVA release 123, available at https://www.arb-silva.de/documentation/release-123/.
